# A Digital Microfluidic Device Integrated with Electrochemical Impedance Spectroscopy for Cell-Based Immunoassay

**DOI:** 10.3390/bios12050330

**Published:** 2022-05-12

**Authors:** Yuqian Zhang, Yuguang Liu

**Affiliations:** 1 Department of Surgery, Division of Surgical Research, Mayo Clinic, Rochester, MN 55905, USA; zhang.yuqian@mayo.edu; 2 Microbiome Program, Center for Individualized Medicine, Mayo Clinic, Rochester, MN 55905, USA; 3 Department of Immunology, Mayo Clinic, Rochester, MN 55905, USA

**Keywords:** digital microfluidics, non-faradic electrochemical impedance spectroscopy (EIS), cell-based immunoassay, interdigitated electrode

## Abstract

The dynamic immune response to various diseases and therapies has been considered a promising indicator of disease status and therapeutic effectiveness. For instance, the human peripheral blood mononuclear cell (PBMC), as a major player in the immune system, is an important index to indicate a patient’s immune function. Therefore, establishing a simple yet sensitive tool that can frequently assess the immune system during the course of disease and treatment is of great importance. This study introduced an integrated system that includes an electrochemical impedance spectroscope (EIS)-based biosensor in a digital microfluidic (DMF) device, to quantify the PBMC abundance with minimally trained hands. Moreover, we exploited the unique droplet manipulation feature of the DMF platform and conducted a dynamic cell capture assay, which enhanced the detection signal by 2.4-fold. This integrated system was able to detect as few as 10^4^ PBMCs per mL, presenting suitable sensitivity to quantify PBMCs. This integrated system is easy-to-operate and sensitive, and therefore holds great potential as a powerful tool to profile immune-mediated therapeutic responses in a timely manner, which can be further evolved as a point-of-care diagnostic device to conduct near-patient tests from blood samples.

## 1. Introduction

A healthy immune system is of great importance in the fight against various diseases, from cancer to infections and auto-immune diseases [1,2]. The immune system often responds dynamically to abnormalities and invaders, and a disease-burdened immune system can provide a window into the disease status and its prognosis. For example, human peripheral blood mononuclear cells (PBMCs, 0.7–6.2 × 10^6^ cells per mL of blood [3]) are the major players in the immune system in the peripheral blood, and they consist of the following few subsets: lymphocytes (B cells and T cells), monocytes, natural killer cells (NK cells), and dendritic cells [4]. Over the recent years, PBMCs have been gaining attention as a promising noninvasive tool to examine the immune system for indicators of therapeutic responsiveness to various critical diseases. The dynamics of PBMC abundance during the course of diseases and treatment, such as cancer therapy, were reported to reflect the clinical tumor burden due to the tumor-immune cell interaction including cytotoxic differentiation and the activation of interferon signaling in peripheral T cells [5]. Therefore, the need for timely assessment of the PBMC population during the course of disease and treatment is mounting. Currently, flow cytometry is the gold standard of PBMC counting due to its proven sensitivity and reliability; however, it relies on highly specialized and bulky equipment in lab facilities and thus is logistically challenging to be used for frequently monitoring the trajectory of the immune functions under an evolving disease status. In addition, it often requires complex sample preparation procedures, including fluorescent labeling (which mostly relies on a panel of eight fluorescent markers and is often challenged by spectral overlap [6,7]) and technical expertise, which adds extra difficulties and costs for implementing them as point-of-care (POC) tests that allow PBMC monitoring on a regular basis to better triage patients for the most suitable care options and allocate medical resources in an effective manner [8]. Therefore, there is an unmet need for developing an easy-to-operate platform that can quantitatively detect PBMCs in a rapid and sensitive manner. 

More recently, digital microfluidics (DMF) has been gaining attention as an ideal candidate for the POC testing platform. Briefly, DMF is a liquid operation technology that allows the manipulation of droplets on a planar electrode surface under the applied voltage based on the electrowetting-on-dielectric (EWOD) principle. In a DMF device, droplets (pico- to micro-liter) can be operated in an automated and programmable fashion with minimally trained hands [9]. In addition, instead of relying on physical microstructures, DMF platforms can be reconfigured on-demand for different applications such as immunological assays, cell-based assays, and DNA amplification, thus making them a versatile tool for POC testing [10,11,12]. Electrical-based detection is one of the most promising sensing strategies that does not rely on microscopy or other advanced optics. This sensing mechanism presented high compatibility with DMF platforms due to the convenience of integrating electrical sensors into systems that are composed of electrode arrays. The merging of electrical-based sensors and DMF platforms enabled various POC applications, most notably immunoassays [13,14]. To date, the integration of electrical sensors into DMF platforms has been reported for the detection of antigen molecules [15,16] and rubella virus [13] with high sensitivity and selectivity. In general, these detections are usually based on enzyme-linked immunoassay (ELISA), which requires immobilizing target molecules on solid surfaces such as magnetic beads, followed by labeling the captured molecules with enzyme-tagged secondary antibodies. In this way, targets can be quantified by measuring their electrochemical oxidation in the presence of redox probes, which can be achieved via amperometric or potentiometric transduction principles with electrochemical electrodes housed on the DMF substrates. Although those transduction techniques can lead to optimal performance in detection, a relatively high applied voltage (~1 V) is often required, which might damage or disturb the bio-recognition events [17,18]. Therefore, electrochemical impedance spectroscopy (EIS), as an alternative electrochemically non-destructive evaluation method, has been widely used in recent years. In EIS, a small sinusoidal AV voltage (V_peak_ = 10 mV–100 mV) is applied to the electrodes to measure the impedance across the electrode-electrolyte interface, presenting the magnitude and the phase as output signals. The targets can be quantified by measuring the change in the impedance signals upon the binding of the molecules on the electrode surface.

In this study, we integrated EIS into a DMF platform and performed a cell-based immunoassay for the quantification of PBMC abundance as the first application. We first conducted finite element method-based simulation on different dimensions of the interdigitated electrodes to obtain optimal detection sensitivity, followed by experimental validation. Moreover, we exploited the unique fluid manipulation property of DMF devices to conduct the cell incubation in a dynamic mode, which achieved 2.4 times signal enhancement compared with the conventional incubation in a stationary mode. This integrated platform provided the following advantages: (1) The automated fluid handling in the DMF platform simplifies the operation procedures, including sample incubation and washing; (2) low sample volume (4 μL) can be used in this integrated system, and the sample droplets can be easily delivered to different sensing electrodes for parallel detection; (3) sample droplets can be operated dynamically on-chip; this, therefore, allows the dynamic incubation of the immunoassay to achieve better sensitivity. Overall, our platform demonstrated great potential as a simple yet sensitive analytic tool to enumerate PBMCs for frequently monitoring immune responses over the course of diseases and treatment.

## 2. Materials and Methods

All chemicals were purchased from Sigma-Aldrich (St. Louis, MO, USA) unless otherwise noted. 

### 2.1. DMF Device Overview

**Figure 1:**
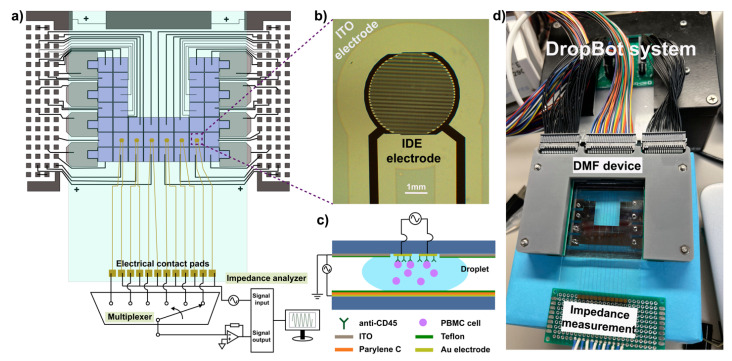
(**a**) Overview of DMF device with integrated interdigitated electrodes (IDEs). Schematic illustration of the device showing 40 actuation electrodes on the bottom plate and 6 sets of sensing electrodes on the top plate. The sensing electrodes were connected to a multiplexer and an impedance analyzer for the detection; (**b**) Microscopic image of IDE electrodes deposited on the top plate. (**c**) Cross-section diagram of the device, with a droplet of cells on the sensing electrodes (not to scale); (**d**) A photo demonstrating the assembly of an integrated DMF device. DropBot system was used to operate the device; the impedance measurement was conducted by connecting electrical contact pads to a digital impedance analyzer.

The overall structure of the DMF device is illustrated in Figure 1a. Briefly, the DMF device is a double-plate EWOD-based device with an array of interdigitated electrodes (IDEs) (Figure 1b) housed on the top plate and the actuation electrodes on the bottom plate. The IDEs were coated with a monoclonal antibody (anti-CD45) to capture target cells (Figure 1c), while the droplets are handled by the actuation electrodes on the bottom. The fabrication procedures of each component are elaborated in the following sections.

### 2.2. Fabrication of the Bottom-Plate 

The layout of the DMF device was designed in AutoCAD (Autodesk, Mill Valley, CA, USA). Overall, the bottom layer of the DMF device contains 8 reservoir electrodes, 40 actuation electrodes (4 mm × 4 mm) and electrical contact pads. The microfabrication procedures of the bottom layer were previously reported by our group [19,20,21]. Briefly, the Cr-coated glass slides pre-coated with an AZ1500 photoresist layer (Telic, Valencia, CA, USA) were covered by a photomask (Cad Art Services, Bandon, OR, USA), exposed in a mask aligner (KLOE, Saint-Mathieu-de-Tréviers, France) and developed in 1:4 MF 351 developer (Kayaku Advanced Materials, Westborough, MA, USA). These substrates were then etched in Cr-Chrome Etch (KMG Electronic Chemicals, Tuscaloosa, AL, USA), followed by immersing in acetone to remove photoresist residue, and finally washed in DI water and dried with N_2_ gas stream. The bottom substrates were then coated with 5 μm Parylene C as dielectric layer using physical vapor deposition (Specialty Coating Systems, Clear Lake, WI, USA), followed by spin-coating (3000 rpm, 30 s) of FluoroPel PFC1101V (Cytonix, Beltsville, MD, USA) as the hydrophobic layer and baking on a hot plate (160 °C, 30 min). 

### 2.3. Fabrication of the Top Layer 

The top plate of DMF device was formed on a 50 mm × 75 mm indium tin oxide- (ITO) coated glass slide (R_S_ = 8–12 Ω m/m) (Delta Technologies Ltd., Stillwater, MN, USA). The top plate housed 6 gold IDEs (Ti 10 nm/Au 100 nm), and each contained 32 fingers with a width of 15 μm, which were used for impedance sensing in parallel. 

The fabrication of the top plate used two photolithography steps followed by etching and lift-off. Prior to the first photolithography step, the ITO substrate was sonicated in acetone for 5 min and then in 2-propanol for 1 min, followed by drying with N_2_ gas. After that, the top plate was spin-coated (3000 rpm, 30 s) with a layer of Shipley S1811 photoresist (Rohm and Haas, Marlborough, MA, USA) and pre-baked on a hot plate (100 °C, 2 min). Next, the top plate covered with a photomask was exposed to UV for 10 s (30 mW/cm^−2^) and developed in MF-321 photoresist developer (Rohm and Haas, Marlborough, MA, USA). The patterned substrate was immersed in the ITO etchant (Fisher Scientific, Hampton, NH, USA) to remove the exposed ITO, thus forming insulating regions for patterning IDEs. The etched substrate was rinsed with DI water and dried with N_2_ gas, then a hydrophobic layer (FluoroPel PFC1101V, Cytonix, Beltsville, MD, USA) was spin-coated (2000 rpm, 60 s) onto the substrate, and the substrate was then treated with oxygen plasma (VLF-1000, Yield Engineering System, Inc., Fremont, CA, USA) to temporarily turn the hydrophobic layer into hydrophilic. 

In the second photolithography step, another layer of S1811 photoresist was spin-coated (3000 rpm, 30 s) onto the substrate, followed by photolithography to define the pattern of the IDE array. When completed, the substrate was cleaned via oxygen plasma to remove surface residues and increase the adhesion between the metal and the glass substrate. Then, a layer of metal (10 nm Titanium/100 nm Gold) was deposited onto the substrate via electron-beam physical vapor deposition (SQC-310, Inficon Bad Ragaz, Switzerland), followed by lift-off technology [22,23]. The substrate was then immersed in acetone to remove the underlying photoresist and was baked (200 °C, 1 h) to restore the hydrophobicity. The dimension of the deposited IDEs was thoroughly checked and evaluated with a Leica optical microscope (DMi 8 Leica, Buffalo Grove, IL, USA). 

### 2.4. Device Assembly and Operation 

In the DMF device assembly, the ITO glass substrate containing an IDE sensing array was used as the top plate and was assembled onto the Cr glass substrate containing actuation electrodes and contact pads as the bottom plate. The two plates were parallelly connected by two layers of double-side electrically conductive tape (127 μm) (3M, Ted Pella, Inc., Redding, CA, USA). The volume of each droplet on the devices was 4 μL. 

The actuation of droplets in the device was operated by a DropBot system [24], which was composed of a fully integrated high-voltage amplifier and a signal generator in a portable box (Figure 1d). The system was connected to a laptop and operated via MicroDrop software. Briefly, droplets were manipulated by applying sine wave potentials (140 V_RMS_, 10 kHz) between the ground electrode (ITO top plate) and actuation electrode (Cr bottom plate) via a Pogo pin electronic interface. Reagents were loaded into reservoirs on the right side of the device via micropipette and dispensed as discrete droplets by switching the voltage “on” and “off” between adjacent electrodes. Droplets were moved across the IDEs for incubation and sensing, and waste fluids were moved to the reservoirs on the left side and then removed with KimWipes (Kimberly-Clark, Irving, TX, USA). 

### 2.5. Surface Functionalization of IDEs 

Prior to the surface functionalization, the Au IDEs were cleaned using an electrochemical reductive cleaning process, by applying a single pulse at +1.4V (DC) for 30 s in PBS buffer [25]. To form a bio-recognition layer on the Au IDEs, the following steps were carried out. First, an insulating thiol carboxyl self-assembled monolayer (SAM) was formed on the electrode surface by incubating in SAM solution (11-Mercaptohexadecanoic acid (MUA) at 1 mM in ethanol) overnight in the dark to form a thiol-sulfide linkage terminated with a COOH function group on the electrode surface. Then, a mixture of N-Hydroxysuccinimide (NHS) and 1-Ethyl-3-(3-dimethylaminopropyl)carbodiimide (EDC) (100 mM NHS and 100 mM EDC prepared in MES buffer (pH 4)) was added onto the SAM surface and incubated for 1 h to activate terminal carboxylic acid groups and form NHS ester. Antibodies (CD45+, 100 μg/mL) were then added onto the electrodes and incubated for 3 h at room temperature. Fourier Transform Infrared (FT-IR) was utilized to verify the surface modification and the conjugation of antibody molecules on the electrode surface. The antibody functionalized electrodes were further incubated with bovine serum albumin (BSA) at 1% *w/v* for 30 min to reduce the uptake of non-specific molecules. 

### 2.6. Characterization of Surface Functionalization 

**Figure 2:**
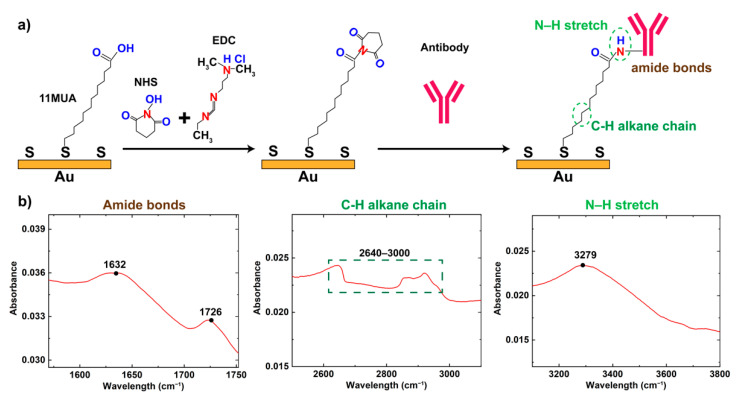
(**a**) Schematic illustrating the formation of SAM layer, EDC/NHS chemistry and antibody conjugation on the gold electrode surface. (**b**) FTIR spectrum for amide bonds, C-H alkane chain and N-H stretch.

Antibodies for capturing PBMCs were immobilized on the gold electrodes. Briefly, SAM was first introduced via thiol-sulfide linkages as depicted in Figure 2a [26,27]. After activating carboxyl-to-amine crosslinking through EDC/NHS chemistry, antibody was covalently conjugated to the SAM layer on the gold surface. The formation of SAM layer was validated by Fourier-Transform Infrared Spectroscopy (FTIR), and the spectra showing amine bonds, N-H stretch, and C-H alkane chain were presented in Figure 2b. The dual absorbance peaks observed between 1726 cm^−1^ and 1632 cm^−1^ were characterized to indicate the presence of amide I and amide II bonds, respectively, after antibody conjugation [28,29]. In addition, FTIR absorbance peak observed at 2925 cm^−1^ indicated the N-H stretch in the formation of covalent link after antibody conjugation, and peak at 3279 cm^−1^ demonstrated the stretching of C-H alkane chain of SAM layer on the gold electrode surface [29]. Table 1 summarized the absorbance peaks extracted from the FTIR spectra in Figure 2b that were associated with the linkers and antibody after immobilizing on the gold electrodes, as compared with the expected absorbance peaks conforming SAM formation. 

### 2.7. Cell-Based Immunoassay 

Impedance detection in the DMF devices was carried out for the following four purposes: (1) to characterize the electrical performance of the IDE array in different conductive solutions to validate the EIS principle; (2) to find out the optimal dimension of IDE array with better sensitivity by characterizing the IDE sensor (15 μm, 30 μm, and 50 μm in “finger” width) response to the PBMCs; (3) to verify the specificity of the EIS sensor by conducting the impedance measurement of blank control and non-specific binding between antibody and cells; (4) to detect PMBCs at different concentrations in stationary mode and dynamic mode. In each experiment, one droplet (4 μL) flowed to the electrodes for impedance measurement. To conduct impedance measurement, the sensing electrodes were interfaced with a multiplexer (Centre Suisse d’Electronique et de Microtechnique) via a pogo pin electronic interface to connect to a multiplexer and digital impedance analyzer (HF2LI/HF2TA, Zurich Instruments, Zürich, Switzerland), which allows up to 6 analytes to be detected in parallel. An AC field with a peak amplitude of 100 mV swept from 100 Hz to 100 kHz, and frequency-based logarithmic sweep mode was used to record the amplitude and phase of the impedance signal to generate the impedance spectrum [30]. Afterward, the data was processed with a custom script written in MATLAB (MathWorks Inc., Natick, MA, USA) for statistical analysis.

First, to verify the EIS principle, PBS at serial dilution concentrations (1M PBS, 0.1 M PBS, 0.01 M PBS, and 0.001 M PBS, containing 0.025% *w/v* Pluronic F-68) were dispensed in the DMF device and the droplets were positioned below the same IDE on the top substrate for EIS measurement. Next, we optimized the geometry of the IDEs to find the optimal structure for better sensitivity. Briefly, the “finger” dimensions of the IDEs were designed to be 15 μm, 30 μm, and 50 μm, while maintaining the same electrode surface area. One droplet of PBMCs in 0.1 M PBS (containing 0.025% *w/v* Pluronic F-68) at the concentration of 10^6^ #/mL was delivered to CD45+ antibody-tagged IDEs and incubated for 20 min at room temperature. After that, three droplets containing wash buffer (0.1 M PBS containing 0.025% *w/v* Pluronic F-68 and 0.05% *w/v* Tween 20) were consecutively flowed across the sensing electrodes to remove unbound cells. The impedance signals were measured before and after the cell incubation. We then compared the impedance changes upon the cell binding (PBMCs in 0.1×PBS at a concentration of 10^6^ #/mL) at three different electrode designs. To verify the sensor specificity, we conjugated IL-6 antibodies on the IDEs, followed by incubation with PBMCs (in 0.1×PBS at a concentration of 10^6^ #/mL). The impedance changes in negative control were compared with signals obtained from IDEs conjugated with CD45+ antibodies that were incubated in pure PBS buffer (no cells, as blank control) and with PBMCs at the same concentration (as target). As a final step, quantitative detection of PBMC was conducted by evaluating the impedance changes at 10^4^ #/mL, 5 × 10^4^ #/mL, 10^5^ #/mL and 10^6^ #/mL. The quantifications were carried out with two modes: (1) stationary mode, which keeps the sample droplet at the sensing area for 20 min; (2) dynamic mode, where the sample droplet was moved following a “forward-backward-backward-forward” pattern (will elaborate in Section 3.3) every 5 min while maintaining the total incubation time for 20 min. Their impedance changes were plotted as a function of cell concentrations on a logarithmic scale, followed by fitting to a linear least square regression. 

## 3. Results and Discussion

During the development of a DMF system integrated with the EIS detection mechanism, one major challenge is the integration of bare sensing electrodes with hydrophilic surfaces into the actuation electrode arrays covered with dielectric and hydrophobic layers. Previous studies have integrated the sensing electrodes on bottom plates [31,32]; however, such integration limits the detection sensitivity since the dielectric layer with low permittivity will impede electrical responses to the analytes. Therefore, integrating sensing electrodes on the top plates has recently become a promising alternative strategy [13,15], as the simpler structure of the top plate makes it easier to be fabricated and replaced. Moreover, the surface area of the hydrophobic ground electrode is much larger compared with the actuation electrodes, thus minimizing the hindrance of droplet movement on the hydrophilic sensing electrodes. For this reason, we integrated an impedance sensing array containing six IDEs on the top substrate first by creating isolated areas in the ITO ground electrode via conventional photolithography, followed by Au electrode deposition at desired regions. 

### 3.1. Equivalent Circuit and Modeling of IDEs

An equivalent circuit, which allows the basic characterization of an electrode-electrolyte system, is commonly built to analyze the electrical behavior of the EIS sensor [31,33,34]. The experimental components were approximated as ideal electronic components such as capacitors and resistors, in order to correlate the overall impedance and change of the system with each component separately, thus finding optimal measurement parameters for the immunoassay. 

**Figure 3:**
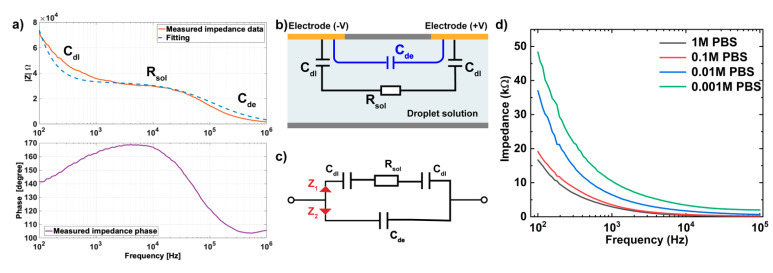
(**a**) The impedance magnitude of IDEs in DMF chip in DI water (non-faradaic regime) and the fitting curve came from Equations (1)–(3) with *C_dl_* = 48 nF, *R_sol_* = 33 kΩ, and *C_de_* = 42 fF. (**b**) Simplified electrical modeling and (**c**) equivalent circuit representing the electrode embedded in a DMF platform when a droplet is present. (**d**) Non-faradic electrochemical impedance spectroscopy (EIS) measured on the IDE in PBS buffer with different concentrations.

The EIS was conducted by measuring the impedance response of IDE in DI water, and the resulting impedance and phase were plotted as a function of frequency from 100 Hz to 1 MHz, as shown in Figure 3a. Here we used the Bode plot for EIS graphical presentation since it associates the applied frequencies with the electronic components of the electrode systems that are non-faradaic and can reflect key information on detection sensitivity [35]. The simplified equivalent circuit of the non-faradaic regime (no redox probe) was typically presented in Figure 3b, containing two parallel branches (Figure 3c) to present the electrode-electrolyte interface and aqueous media. $C_{dl}$ represents the double-layer capacitance formed at the interface of the electrode-electrolyte is due to the accumulated electrical double layer (EDL) under the applied voltage. The aqueous media has both resistive and capacitive responses, and it is presented as solution resistance $R_{sol}$ and solution dielectric capacitance ($C_{de}$) connected in parallel. The total impedance of the system (*Z*) as a function of frequency (f) is noted in Equations (1)–(3). The EIS data were fitted using the equivalent circuit shown in Figure 3b.

(1) $$\frac{1}{\left|Z\right|}=\frac{1}{\left|Z_{1}\right|}+\frac{1}{\left|Z_{2}\right|}$$

(2) $$\left|Z_{1}\right|=\sqrt{R_{sol}{}^{2}+\frac{1}{{\left(2\pi fC_{dl}\right)}^{2}}}$$

(3) $$\left|Z_{2}\right|=\frac{1}{2\pi fC_{de}}$$

Note that the simplified equivalent circuit assumes that the double layer has an ideal capacitance and neglects the non-ideal capacitive characteristics including chemical heterogeneities [33,36], while the ionic double layer in the solid electrode interface has been empirically demonstrated to have a non-ideal capacitive behavior (frequency-dependent) [37,38]. Therefore, we speculate that the impedance mismatch at f< 10^3^ Hz between the measured impedance data and fitting data is caused by the non-ideal characteristics of the experimental system. Meanwhile, the stray capacitance (Cstray), which exists between two proximal electrodes at different potentials, can lead to the storage of opposite electric charges on electrodes [39] and thus cause the observed deviation of the measured data at a high-frequency range (f> 10^5^ Hz).

As depicted in Figure 3a, there are three distinct regions based on frequency ranges, which correspond to the three electronic elements in the equivalent circuit, where double layer capacitance ($C_{dl}$), media resistance ($R_{sol}$) and solution dielectric capacitance ($C_{de}$) are dominant in low-, intermediate-, and high-frequency ranges, respectively. Since the bio-recognition events mainly occurred on the surface of the electrode, which overlapped with the electrical double layer, leading to the change in capacitance $C_{dl}$ [33,40]. Therefore, the EIS measurement was focused on the variation of the $C_{dl}$. At low and intermediate frequency range (f< 10^5^ Hz), $C_{dl}$ and $R_{sol}$ determined the system impedance, since the current cannot flow through the dielectric capacitance and therefore $Z_{2}\;$(Figure 3c) acted as an open circuit, presenting minimal effect on the impedance detection. On the other hand, given the noise and instability signal acquired at a frequency lower than 100 Hz (data not shown) as well as the slow scanning rate at a low-frequency range, 100 Hz was selected as the lower limit of frequency for the detection of $C_{dl}$ variations. Therefore, we further characterized the electrical performance of IDE in PBS solution at various concentrations (1M, 0.1M, 0.01M, and 0.001M PBS) at the frequency range from 100 Hz to 100 kHz, with a focus on the impedance responses to $C_{dl}$ and $R_{sol}$. The non-faradic EIS measurements were taken by moving the sample droplet to the same IDE, and their impedance results were plotted as a function of frequencies in Figure 3d. This result demonstrated the higher impedance observed in PBS with lower concentration due to the reduced $C_{dl}$ (proportional to the electrolyte concentration based on Helmholtz models [41,42]) and increased $R_{sol}$ (inversely proportional to the electrolyte conductivity), which indicated the dominant effects of double-layer capacitance ($C_{dl}$) and solution resistance ($R_{sol}$) in defining sensor impedance response at low- and intermediate-frequency ranges. Moreover, the impedance measurement at a lower frequency is more dominated by the response of $C_{dl}$ than $R_{sol}$ based on the observation in Figure 3a. Therefore, 100 Hz was selected as the optimal frequency for detecting the change of $C_{dl}$ in our EIS system. 

### 3.2. Electric Field Distribution of IDE in DMF Platform

In EIS-based IDEs, the electric field distribution between electrodes plays a vital role in determining the electrical impedance change upon the cell binding on the electrode surface, thus the detection sensitivity. Previous reports have noted the improved detection sensitivity of smaller electrodes due to the confined electric field at the sensing region [34,43]. In order to find the optimal design for a cell-based assay, we designed IDEs with different dimensions and simulated the electric field generated from these electrodes using the finite element method (FEM) in COMSOL Multiphysics 6.0 software (Burlington, NJ, USA).

**Figure 4:**
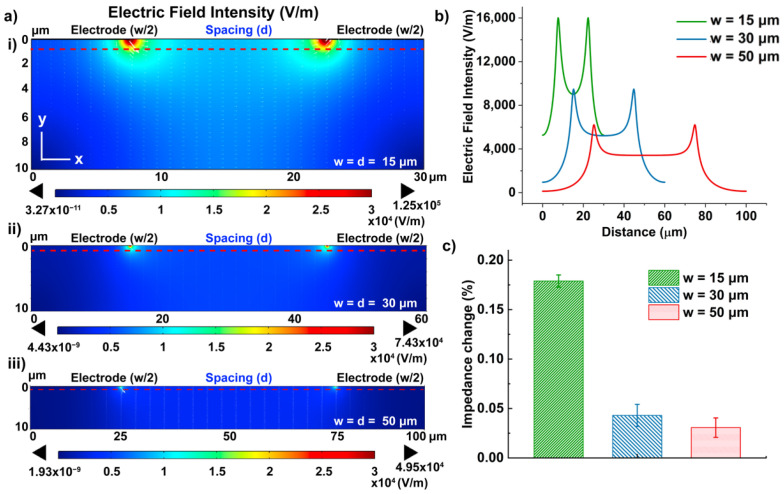
(**a**) 2D cross-section simulation of electric field distribution of IDEs at (i) 15 μm; (ii) 30 μm; (iii) 50 μm; (**b**) Electric field intensity at 1 μm below the electrode surface (corresponding to red dotted lines in (**a**)); (**c**) Experimental analysis of the impedance measurement performance in terms of the dimension of the IDEs, with error bars denoting standard deviation of n = 3.

The simulated 2-D cross-section included a gold electrode on the glass substrate and the ambient fluid (0.1 × PBS solution). To reduce complexity, the model included only two fingers of the replicating electrode structure with opposite voltage polarity [34,44] (Figure 4a). The dimensions of the electrode were defined as width (w = 15 μm, 30 μm, and 50 μm), spacing (d = 15 μm, 30 μm, and 50 μm), and height (h = 100 nm). The frequency-domain electric current module was carried out in the simulation with the following equations below:

(4) $$\nabla \times J=Q_{j,v}$$

(5) $$J=\sigma E+j\omega D+J_{e}$$

(6) E=−∇V

where J represents current density, and $J_{e}$ is the external generated current. $Q_{j,v}$ depicts the change, and E represents the electric field intensity. The dielectric model $D=\varepsilon_{0}\varepsilon_{r}\times E$, where $\varepsilon_{0}$ and $\varepsilon_{r}$ the electrical permittivity of the free space and the medium. ω depicts the angular frequency of the applied AC, and V is the applied voltage. Current conservation was applied to the whole domain, and the outer boundaries were adopted as electric insulation. Voltage terminals were applied to the electrodes (−100 mV and 100 mV), respectively. The electrodes were meshed with 0.003 μm as the minimum element size and 0.2 μm as the maximum element size, and the maximum element growth rate was specified to be 1.3. The rest domain was meshed with free triangular elements using the automatic tessellation method. 

Figure 4a shows the electric field intensity near the electrode region (10 μm below the electrode surface), where the binding between the cells and immobilized antibodies is expected. The simulation demonstrated that the highest electric field was confined at the edge of the electrodes and decayed along the *y*-axis. The electric field intensity at smaller electrode dimensions (peak intensity: 12.5 kV/m for 15 μm) was found to be higher than the larger dimensions (peak intensity: 7.43 kV/m for 30 μm; 4.95 kV/m for 50 μm) (Figure 4a ii and iii). The values of the electric field intensity were taken at 1 μm below the electrode surface (red dash line) along the *x*-axis for a more quantitative comparison among the three designs, where the bound cells start to affect the impedance measurement [33]. The average electrical field intensity of the 15 μm design (9.52 kV/m) was calculated to be 2.2 times and 4 times higher than the 30 μm (4.32 kV/m) and 50 μm (2.39 kV/m) designs, consequently. Therefore, we expected the IDEs with a 15 μm electrode dimension would have the optimal sensitivity for cell detection. 

To further demonstrate the correlation between the electrode dimension and the detection sensitivity, cell detection was performed on IDEs with different dimensions embedded in the top plate of the DMF devices. Though their finger sizes were different, the total electrode area for probe immobilization remained the same. After the conjugation of anti-CD45 antibodies on the electrode surface, droplets containing PBMCs in 0.1M PBS (10^6^ #/mL) were moved to the IDEs and incubated at room temperature for 20 min. Considering the batch effect on electrode geometry during microfabrication [45] as well as the stochastic nature of the chemical reaction, which results in deviation in the number of antibodies conjugated to the surface [46,47], the impedance changes were thus analyzed by taking a relative measurement before and after cell binding to the surface to cancel out those variances. Therefore, the impedance signals measured from the antibody functionalized (after BSA blockage) at 100 Hz as baseline ($Z_{base}$), and the impedance change after cell incubation ($Z_{cell}$) were quantified using the following equation: $Impedance\;change\;\left(\%\right)=\frac{Z_{cell}-Z_{base}}{Z_{base}}\times 100$, similar to other reported work [48,49]. Results shown in Figure 4c demonstrated the impedance changes upon the cell incubation at IDEs with three different dimensions, and the impedance change measured at an IDE with a width of 15 μm was >3-fold higher than the same assay using 30 μm and 50 μm-wide IDEs, thus presenting better detection sensitivity.

### 3.3. Peripheral Blood Mononuclear Cells (PBMCs)-Immunoassay on DMF Chips

**Figure 5:**
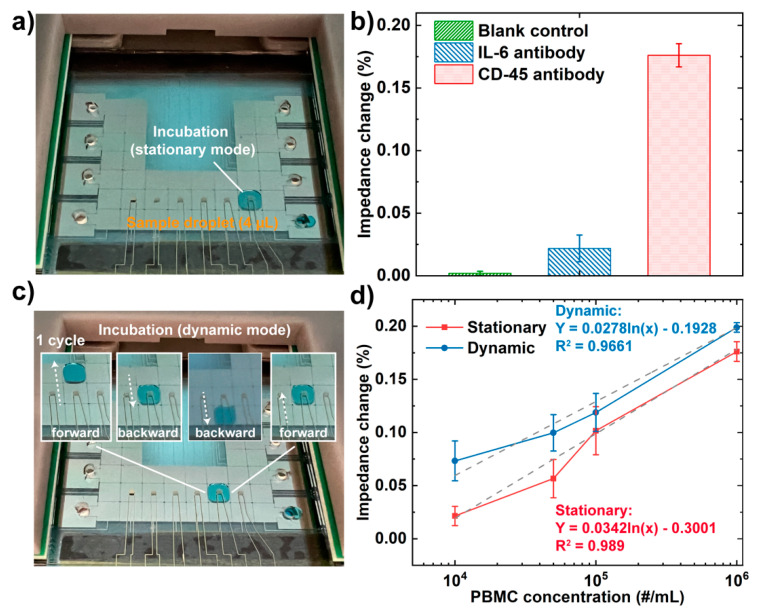
Photos of (**a**) Droplet incubation in the stationary model; (**b**) Impedance changes of CD-45 antibody against PBS buffer (no cell present, blank control), IL-6 antibody against PBMCs (negative control), and CD-45 antibody against PBMCs (target). The concentrations of the cell suspensions were 10^6^ per mL. (**c**) a droplet trajectory in the dynamic incubation mode (1 cycle); (colored fluid was used for better visualization); (**d**) The relationships between the concentration of PBMCs and the corresponding impedance changes of the integrated EIS sensor in stationary and dynamic modes. The error bars represent the standard deviation of n = 5.

To test the level of non-specific binding in this approach, we performed two control experiments. First, we conducted a blank control by incubating CD45 antibody IDE with PBS buffer without any cells present. Second, we conjugated IL-6 antibodies onto the IDEs to test whether PBMCs can be non-specifically captured. We compared the results with a target case using the CD45 antibody conjugated IDE to capture PBMCs under the same condition (Figure 5a). It was found that the impedance change caused by non-specific absorption (2.2%) was much lower than that of specific capture (17.6%) in Figure 5b. The blank control presented a signal variation of only 0.19% during the impedance measurement.

Further, to evaluate the use of EIS-based DMF devices in numerating cells, a PBMC-assay was developed to quantitatively detect the concentration of cells by recognizing their specific cell surface marker CD45. The detection was achieved by first immobilizing CD45 antibody on the IDE, followed by moving droplets containing PBMCs in a series of concentrations (10^4^/mL, 5×10^4^/mL, 10^5^/mL, and 10^6^/mL) to different IDEs and evaluate the impedance changes. 

A previous study reported by Wheeler’s group demonstrated the enhanced binding kinetics between the target antibody and the immobilized antigen in dynamic mode (by moving droplets to pass the antigen spot multiple times during hybridization), and this can also reduce sample processing time and non-specific binding [50]. Based on this, we have implemented dynamic detection in our impedance measurement system, expecting that increased binding kinetics can improve the detection sensitivity. To achieve dynamic incubation, the droplet containing PBMCs was moved following a “forward-backward-backward-forward (1 cycle)” pattern on the detection electrode every 5 mins’ incubation (Figure 5c). Meanwhile, a stationary mode was carried out by keeping the droplet unmoved below the detection electrodes during the incubation as a control (Figure 5a). The detection performance of the two modes was compared in terms of detection sensitivity. As shown in Figure 5d, the impedance changes obtained after PBMC incubation at different concentrations using both modes can be fitted by a logarithmic regression equation, and the impedance changes in the dynamic incubation model were higher compared with the stationary model. The impedance increments ($Impedance\;increment\;\left(\%\right)=\frac{Z_{dynamic}-Z_{stationary}}{Z_{stationary}}\times 100$) at different cell concentrations were compared in Table 2, ranging from 12.9% to 242.7%.

In addition, it is interesting to observe that the impedance increments of the impedance signal measured in the dynamic mode were over 20 times higher at a low concentration (10^4^ # cells/mL) than at a high concentration (10^6^ # cells/mL). Since a stationary droplet in the DMF device was presented as no flow [50,51], the molecular binding in the stationary mode mostly occurred at the interface of the electrode surface. Therefore, only a small portion of the fluid was in contact with the electrode surface, and thus only the cells near the interface were able to bind with the immobilized antibodies. On the contrary, in the dynamic incubation mode, the droplet was moved back and forth on three adjacent actuation electrodes. A theoretical study reported by Kim’s group indicated that two circulating internal flows are present within a droplet along its advancing direction [51]. Therefore, we speculate that the circulating flows in the dynamic incubation mode can enhance the possibility of cells interacting with antibodies, leading to a higher binding efficiency. In addition, we expect the localized depletion of cells after binding will be rapidly replenished in the dynamic mode due to the relatively small sensing area (1.57 × 10^6^ μm^2^) compared with the droplet (16 × 10^6^ um^2^), therefore permitting constant cell binding with the antibody on the sensing electrode. On the other hand, the cell binding capacity was estimated based on the surface area of the electrode surface (1.57 × 10^6^ μm^2^) divided by the average cross-section area of PBMC (41.85 μm^2^, assuming the average diameter of PBMC is 7.23 μm [52]), and the estimated maximum capacity of cells is 3.76 × 10^4^. Though the actual binding capacity will be less than the estimated maximum capacity given the steric effect between cells [53], the cell assay at the highest concentration (10^6^ per mL) was below the saturation level (~4 × 10^3^ cells in a 4 μL droplet). Therefore, the dynamic movement of cells in a droplet at the highest concentration still presented an enhanced impedance signal (11.3%). As for cells at lower concentrations (10^4^ per mL), the dynamic incubation greatly enhanced the cell binding efficiency and, consequently, higher impedance change over two times. 

## 4. Conclusions

In this work, for the first time, we have developed a DMF platform integrated with an EIS-based biosensor with bare sensing electrodes for the detection of PBMCs. IDEs were optimized for improved detection sensitivity. The measured impedance spectra demonstrated good agreement with the theoretical impedance spectra based on the equivalent circuit. In addition, the correlation between electrode dimension and the electric field intensity showed an over 3-fold sensitivity improvement on the 15 μm finger-sized IDE compared with 30 μm and 50 μm-wide IDEs. Further, the integrated platform was utilized to detect PBMCs at different concentrations in stationary and dynamic cell incubation modes. Higher sensitivity was observed in dynamic mode, whereas low as 10^4^ PBMCs per mL was detected, which is approximately two orders of magnitude less than their biologically relevant range. Overall, the integrated system presented the technical feasibility of detecting immune cells in a simple and sensitive manner. Moreover, due to the feasibility of operating droplets continuously on DMF devices, our future goal is to extend the application to phenotyping immune cell subtypes from a single droplet of a blood sample by passing it through multiple electrodes conjugated with different antibodies. Currently, the mass production of this platform remains a challenge due to its complex microfabrication procedures in cleanroom settings. However, with the rapid advances in novel microfabrication technologies, it is foreseeable that the platform can be mass-produced at a low cost on flexibles using advanced high-resolution printing technologies. We envision that this integrated platform will be useful for evaluating treatment strategies that require frequent assessment of patients’ immune systems as a POC testing platform outside of a centralized laboratory. 

## Figures and Tables

**Table 1:** FTIR peaks confirming SAM formation [28,29].

Description	Expected Peak Position (cm^−1^)	Observed Peak Position (cm^−1^)
Amide I and II bonds	1470–1800	1632, 1726
N–H stretch	3225–3280	3279
Stretching of C-H alkane chain	2640–3000	2925

**Table 2:** Comparison of impedance increment (dynamic vs. stationary modes) at different PBMC concentrations.

PBMC Concentration (#/mL)	10^4^	5 × 10^4^	10^5^	10^6^
Impedance Increment (%)	242.7%	64.4%	26.9%	12.9%

## Data Availability

Not applicable.
